# Do future healthcare professionals advocate for pharmacogenomics? A study on medical and health sciences undergraduate students

**DOI:** 10.3389/fphar.2024.1377420

**Published:** 2024-04-11

**Authors:** Hanan Al-Suhail, Mohammad Omar, Majd Rubaeih, Tamer Mubarak, Margarita-Ioanna Koufaki, Ioannis Kanaris, Favio Mounaged, George P. Patrinos, Maha Saber-Ayad

**Affiliations:** ^1^ College of Medicine, University of Sharjah, Sharjah, United Arab Emirates; ^2^ Department of Pharmacy, Laboratory of Pharmacogenomics and Individualized Therapy, School of Health Sciences, University of Patras, Patras, Greece; ^3^ United Arab Emirates University, College of Medicine and Health Sciences, Department of Genetics and Genomics, Abu Dhabi, United Arab Emirates; ^4^ United Arab Emirates University, Zayed Center for Health Sciences, Abu Dhabi, United Arab Emirates; ^5^ Erasmus University Medical Center, Faculty of Medicine and Health Sciences, Department of Pathology, Clinical Bioinformatics Unit, Rotterdam, Netherlands

**Keywords:** ADR, adverse drug reaction pharmacogenomics, undergraduate students, questionnaire, attitudes, intentions to adopt

## Abstract

Pharmacogenomics (PGx) is a rapidly changing field of genomics in which healthcare professionals play an important role in its implementation in the clinical setting, however PGx level of adoption remains low. This study aims to investigate the attitude, self-confidence, level of knowledge, and their impact on health sciences undergraduate students’ intentions to adopt PGx in clinical practice using a questionnaire developed based on the Theory of Planned Behavior (TPB). A model was proposed and a questionnaire was developed that was distributed to 467 undergraduate students of all academic years from four different departments of the University of Sharjah (UoS) including medical, dental, nursing, and pharmacy students from September 2022 to November 2022. Descriptive statistics along with factor analysis and regression analysis were conducted. The proposed model had a good internal consistency and fit. Attitude was the factor with the greatest impact on student’s intentions followed by self-confidence and barriers. The level of knowledge had a meaningless impact. The majority of students shared a positive attitude and were aware of PGx benefits. Almost 60% of the respondents showed a high level of knowledge, while 50% of them were confident of implementing PGx in their clinical practice. Many students were prone to adopt PGx in their future careers. PGx testing cost and the lack of reimbursement were the most important barriers. Overall, students shared a positive intention and were prone to adopt PGx. In the future, it would be important to investigate the differences between gender, year of studies, and area of studies studies and their impact on students’ intentions.

## Introduction

Pharmacogenomics (PGx) is a scientific discipline that merges pharmacology and genomics (Abdela et al., 2017). PGx testing is becoming more and more widely available in healthcare settings, and a growing body of actionable high-level evidence for clinical utility mandates the provision of sustainable PGx education to healthcare professionals. As a component of personalized medicine, PGx uses genetic information to optimize therapeutic benefits, enhance clinical outcomes, and reduce drug adverse reactions (ADR) (Klein et al., 2017; Barker et al., 2022). Thus, PGx can potentially improve the therapeutic strategy of a patient by selecting the appropriate medication at the correct dosage (Verbelen et al., 2017). Although, sub-optimal response to medication along with the presence of ADRs can be partially explained by other factors including sub-dosing, drug allergy, drug-drug interactions, or lack of patient compliance, a person’s genetic makeup remains an important factor to consider (Rollinson et al., 2020). Many clinical studies in adult patients have demonstrated the clinical utility of PGx in drug management (Klein et al., 2017; Verbelen et al., 2017; Swen et al., 2023).

PGx applications have gained momentum in the last decades thanks to the completion of the Whole Genome Sequence and AllOfUs program in the United States, and led to an increased popularity and the launch of other clinical projects such as the Emirati Genome Project in the United Arab Emirates (UAE) (Klein et al., 2017; Al-Ali et al., 2018). Healthcare professionals including physicians, pharmacists, dentists, and nurses are welcoming the PGx concept and are trying to incorporate it into their clinical practice but at a slow pace (Hansen et al., 2022). Their role in PGx clinical application has been thoroughly investigated in many studies (Albassam et al., 2018; Algahtani, 2020; Alhaddad et al., 2022). Indeed, it was shown that healthcare professionals had a rather positive attitude and were willing to adopt it but they lacked the proper knowledge and training along with self-confidence (Abdela et al., 2017; Albassam et al., 2018; Algahtani, 2020; Smith et al., 2020; Albitar and Alchamat, 2021; Alhaddad et al., 2022; Hansen et al., 2022; Hayashi and Bousman, 2022).

Several challenges and barriers impede PGx’s widespread implementation despite the proven clinical and economic effectiveness of PGx (Chenoweth et al., 2020; Koufaki et al., 2021). The lack of healthcare knowledge and training in the field, the high cost of PGx testing, the lack of reimbursement, and moral and bioethical concerns are some examples. This slow PGx adoption rate in clinical practice must change. To do so, future healthcare professionals must receive a proper education in PGx aiming to overcome their concerns and reluctance. There is another publication, by Rahma and coworkers, 2020, about UAE undergraduate and postgraduate students’ attitudes and level of knowledge related to genomics and PGx (Rahma et al., 2020). In contrast to Rahma and coworkers, 2020 study, our project concentrated on undergraduate medical and health science students and it investigates the impact of four different factors in their intention to adopt PGx and not only two (Rahma et al., 2020). In addition, the survey instrument used in this project was developed based on a behavioral theory and not only based on literature.

In this study, we investigated the attitudes, self-confidence, level of knowledge, barriers, and intentions of health sciences undergraduate students from different Colleges of the University of Sharjah (UoS) to adopt PGx applications in clinical practice using a questionnaire developed based on Theory of Planned Behavior (TPB). Our objectives were to evaluate the impact of different factors on students’ intentions and to highlight any correlations or relations among factors.

## Materials and methods

### Research framework

**FIGURE 1:**
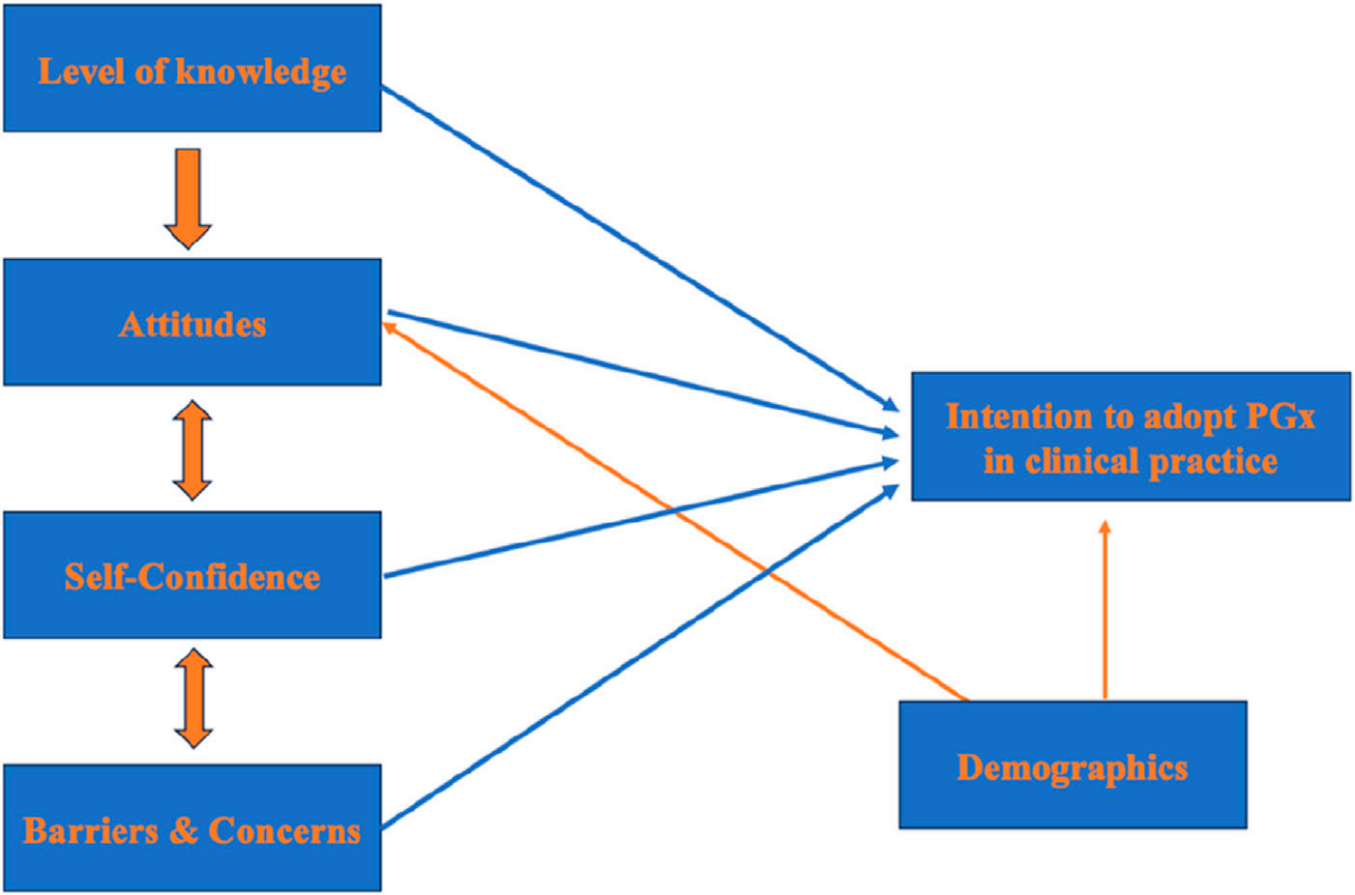
The framework of the proposed model on which the study’s survey was based.

Based on the theory of TPB, we created a modified framework for assessing the effect of several variables on health science students’ intention to adopt PGx testing in their clinical practice. This behavioral theory enables to investigate of the correlation of beliefs, attitudes, and intentions to a behavior since it assumes behavioral intention is the key determinant of behavior (U.S. Department of Health and, Health, 2012). TBP pinpoints that three main factors affect a person’s intention; normative beliefs (attitudes), social influence and beliefs (subjective norm), along with control beliefs (perceived behavior control) (Bosnjak et al., 2020). In parallel, it is assumed that other external factors do not independently affect a person’s behavior (Godin and Kok, 1996; Bosnjak et al., 2020). In our case, we included four factors including attitude (attitudes, compatibility of PGx, PGx clinical benefits), level of knowledge, self-confidence/self-efficacy, barriers, and concerns along with one moderator (demographics). The proposed model along with the factors’ relationships are depicted in Figure 1.

### Study design

A descriptive cross-sectional survey was conducted from September 2022 to November 2022. This study used a validated 41-item questionnaire developed by the Laboratory of Pharmacogenomics and Individualized Therapy at the Department of Pharmacy, University of Patras, Greece, and previously published (Siamoglou et al., 2021a; Siamoglou et al., 2021b; Koufaki et al., 2022). The questionnaire was written in English. It consisted of six main sections that included demographics (5 questions), general knowledge related to PGx interventions (11 questions), attitudes (6 questions), self-confidence in applying PGx in a professional setting (6 questions), barriers and concerns (7 questions), willingness to adopt PGx in clinical practice (6 questions). All items were measured on a seven-point Likert scale, with one being “totally disagree” and seven being “totally agree”. Only the knowledge section was measured on a three-point scale ranging from (agree, disagree, and not sure). The study was approved by the UoS Research Ethics Committee (REC-22-06–06-01-S). An informed consent was provided, and participants had to give their approval before proceeding with the questionnaire’s distribution.

### Study sample

The study sample consisted of 467 undergraduate students from four different departments of UoS including medical, dental, nursing, and pharmacy students. An online questionnaire was distributed via Google Forms to all enrolled undergraduate health science students of all academic years. Students could participate only one time using their academic email, while they could update their answers before final submission. Almost two-thirds of the sample were female students as expected based on students’ representation in each department, since almost 70% are women. Moreover, 35% of participants derived from the Department of Medicine. Participants were dispersed equally, although representation from first and fifth year students was low. The multinational environment of UoS was illustrated in the examined cohort as well. Indeed, students from 35 different nationalities were included in the study, with 17% derived from Syria, 15.4% from Jordan, 14.3% from Egypt, 11% from the United Arab Emirates, and 13% from other countries. Countries with students’ representation of less than 10 students per country were grouped into one named “others”. The following countries were included: Afghanistan, Algeria, Australia, Bahrain, Bangladesh, Canada, Comoros, Djibouti, Dominica, Finland, India, Iran, Japan, Kenya, Kuwait, Lebanon, Mauritania, Morocco, Nigeria, Oman, Pakistan, Philippines, Saudi Arabia, Spain, Sri Lanka, Sweden, USA. Finally, only 135 out of 467 students had attended a PGx lecture in the past. Table 1 summarizes the demographics of the sample.

**TABLE 1:** Students’ demographics.

Gender (%)	
All	467
Male	114 (24.4%)
Female	353 (75.6%)
Department (%)	
Medicine	172 (36.8%)
Dentistry	75 (16%)
Nursing	106 (22.7%)
Pharmacy	114 (24.5%)
Year of Studies (%)	
1st year	60 (12.9%)
2nd year	131 (28%)
3rd year	103 (22%)
4th year	99 (21.2%)
5th year	74 (15.9%)
Nationality (%)	
Egypt	67 (14.3%)
Iraq	35 (7%)
Jordan	72 (15.4%)
Kuwait	33 (7%)
Palestine	24 (5%)
Somalia	14 (3%)
Sudan	16 (3%)
Suria	80 (17%)
UAE	51 (11%)
Yemen	13 (3%)
Other^a^	62 (13%)
Have you attended any PGx- related lectures? (%)	
Yes	135 (29%)
No	332 (71%)

^a^ Includes countries from which there were less than 10 students.

### Data analysis

SPSS statistical tool (version 28; IBM, NY, USA) was used. Frequencies, the proportion of correct replies, descriptive statistics (mean value, standard deviation (SD)), and regression analysis were included in the data analysis. Factor analysis using Cronbach’s Alpha Analysis was used to assess the integrity of the scale of our five factors questionnaire including demographics, level of knowledge, attitudes, self-confidence, and willingness to adopt PGx in clinical practice. All these are illustrated in graphs. Goodness-of-fit tests such as the Chi-square test, Comparative Fit Index (CFI), Goodness of Fit Index (GFI), Tucker-Lewis Index (TLI), Root Mean Square Error of Approximation (RMSEA) were also used to confirm the survey’s validity and reproducibility.

## Results

**FIGURE 2:**
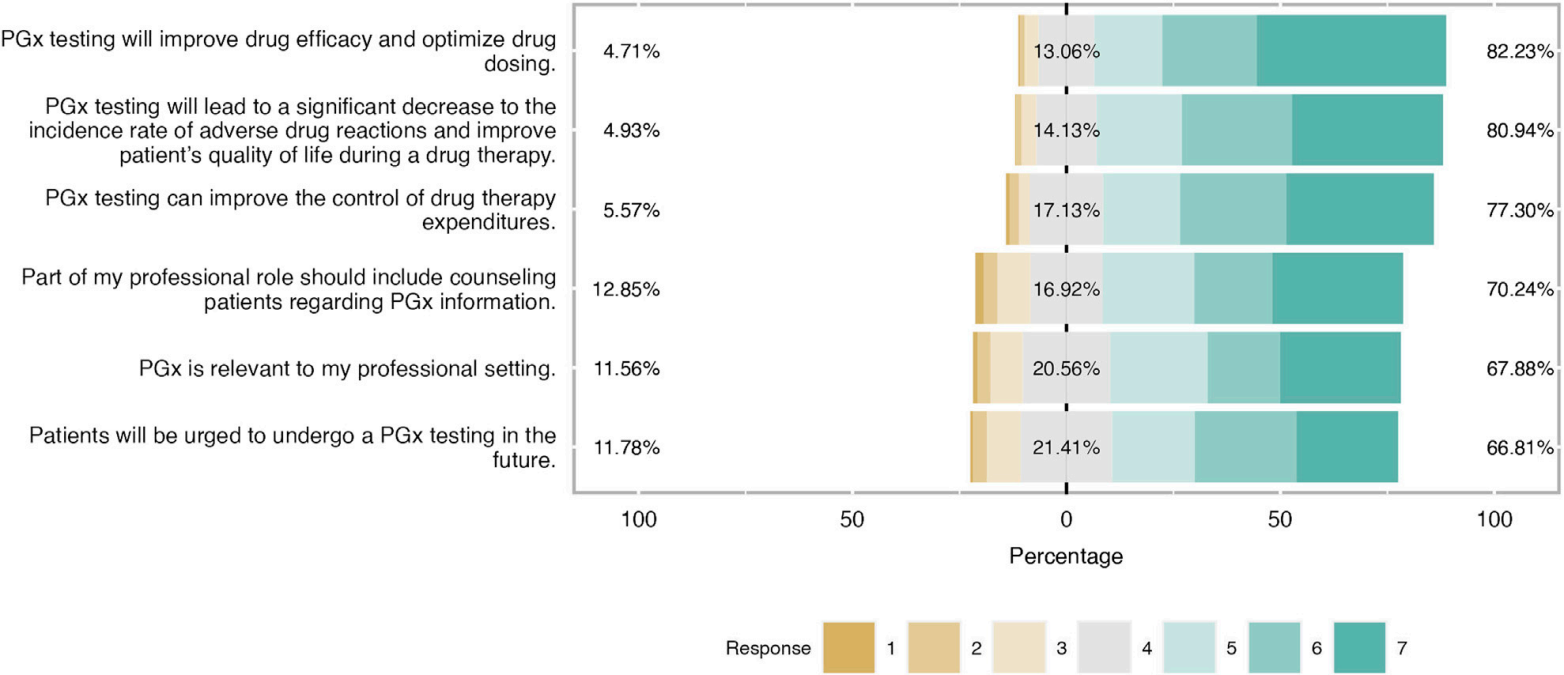
Students’ attitudes towards PGx clinical application. As it is shown in the bar plot, the majority of students have a positive attitude towars PGx application in the clinical practice and were aware of PGx benefits.

**FIGURE 3:**
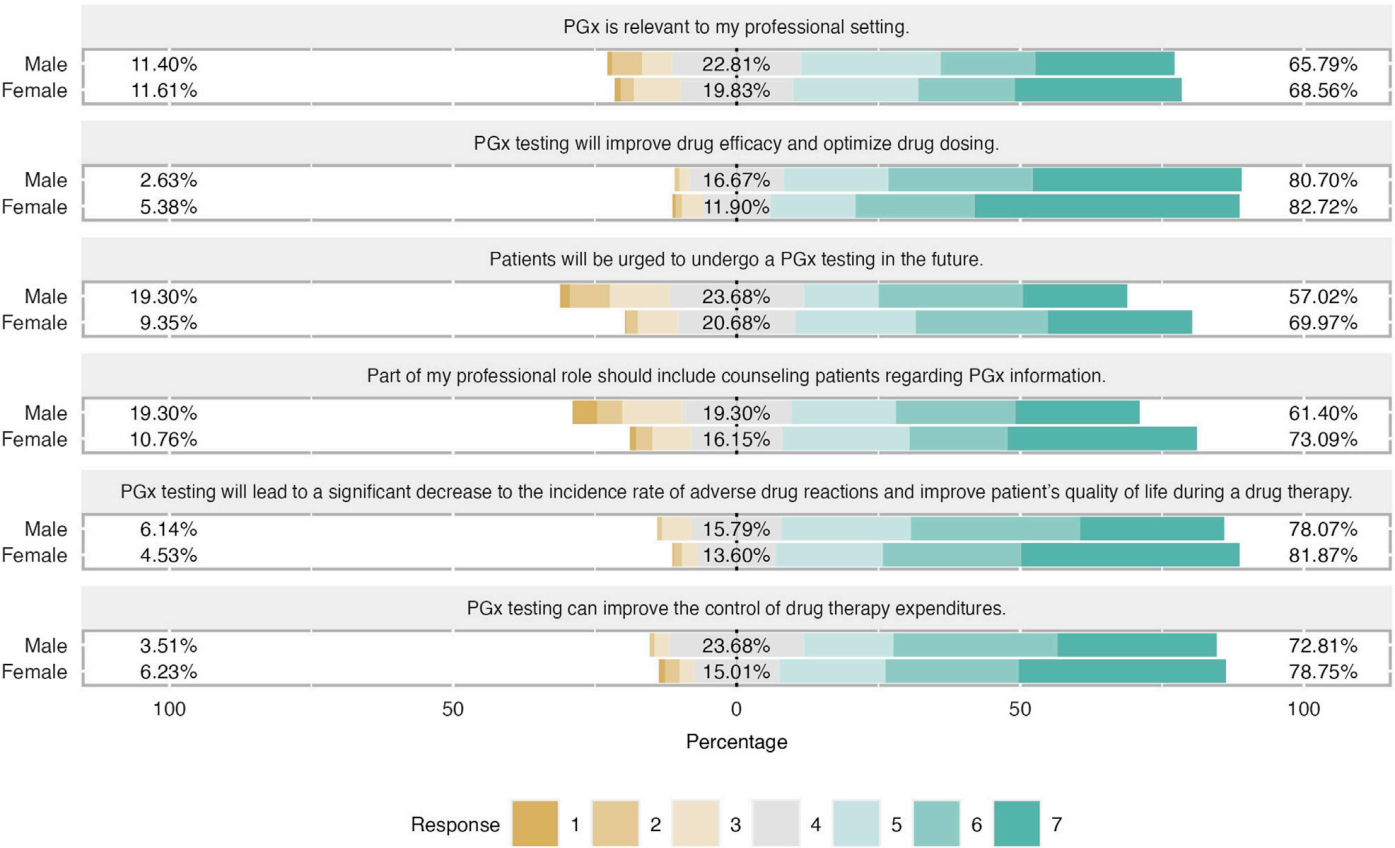
Differences in attitudes based on students’ gender. Female students has slightly more positive attitude regarding PGx testing in clinical practice. Almost 75% of female students claimed that counseling patients for their PGx results is relevant of their profesion and they believed that patients will do a PGx testing in the future.

**FIGURE 4:**
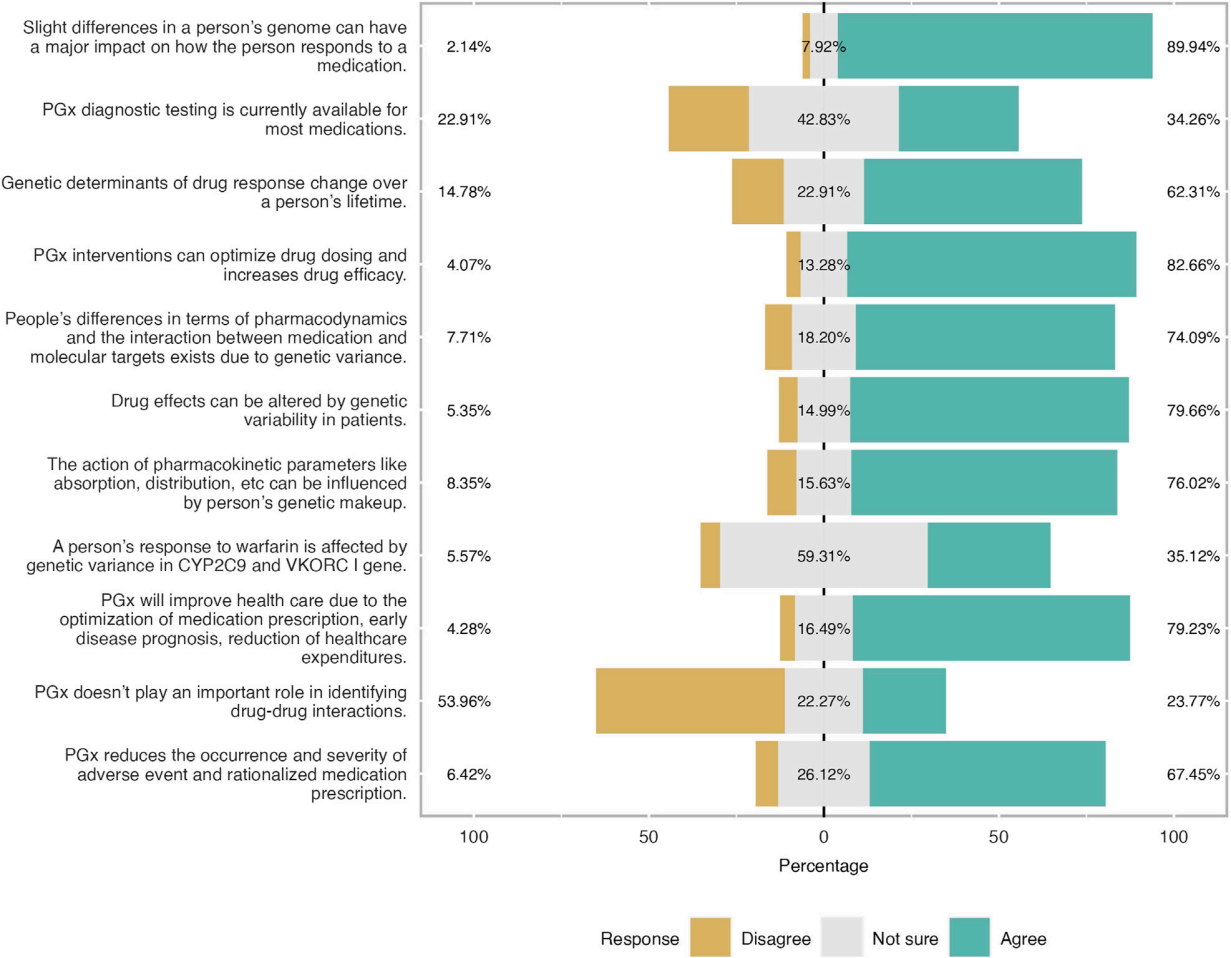
Level of knowledge as reported by participants expressed in three-level Likert scale. Overall respondents had a good level of knowledge especially the theoretical ones. 83% of the students were aware that PGx will optimise drug dosing and improves drug efficacy.

**FIGURE 5:**
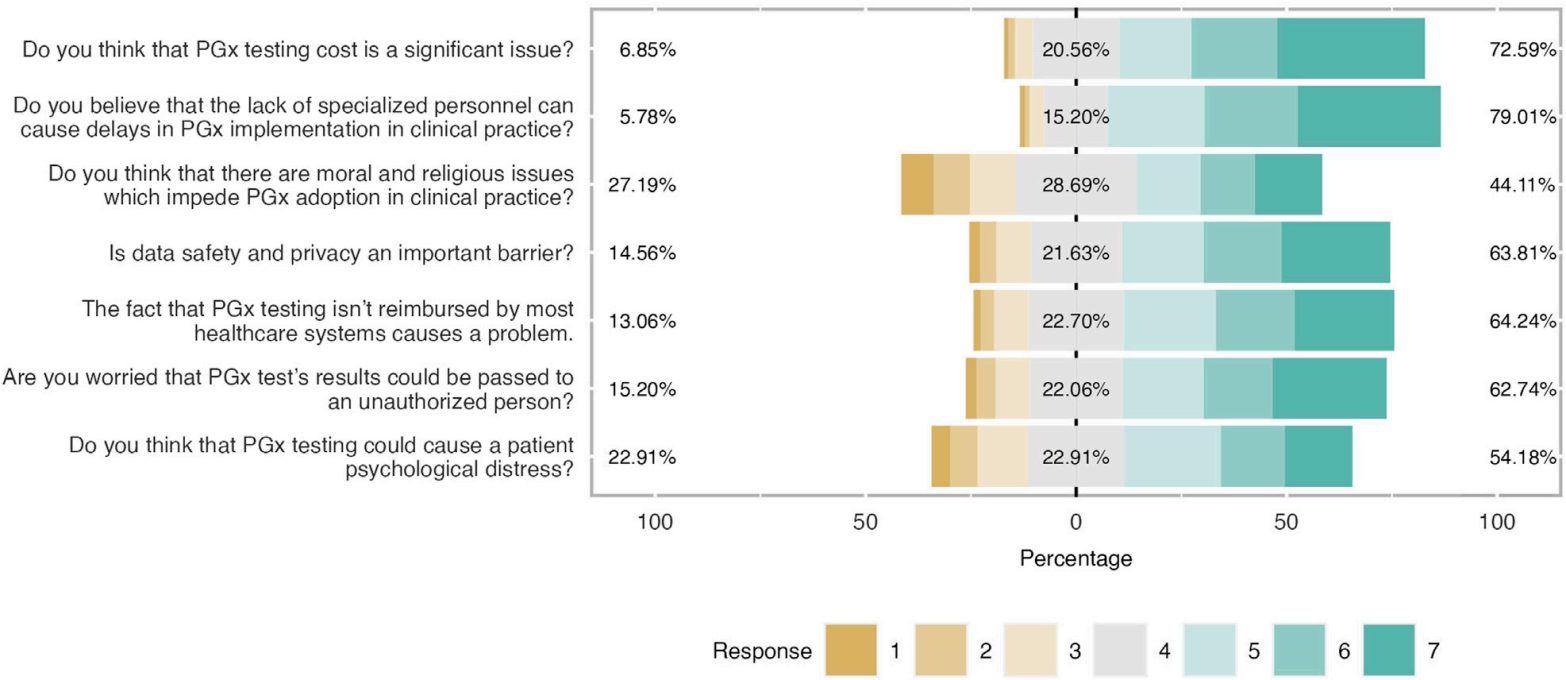
Main barriers and concerns about PGx clinical application. Respondents agreed with most noted barriers and concerns. It was shown that there were not important moral and religious issues related to PGx implementation.

**FIGURE 6:**
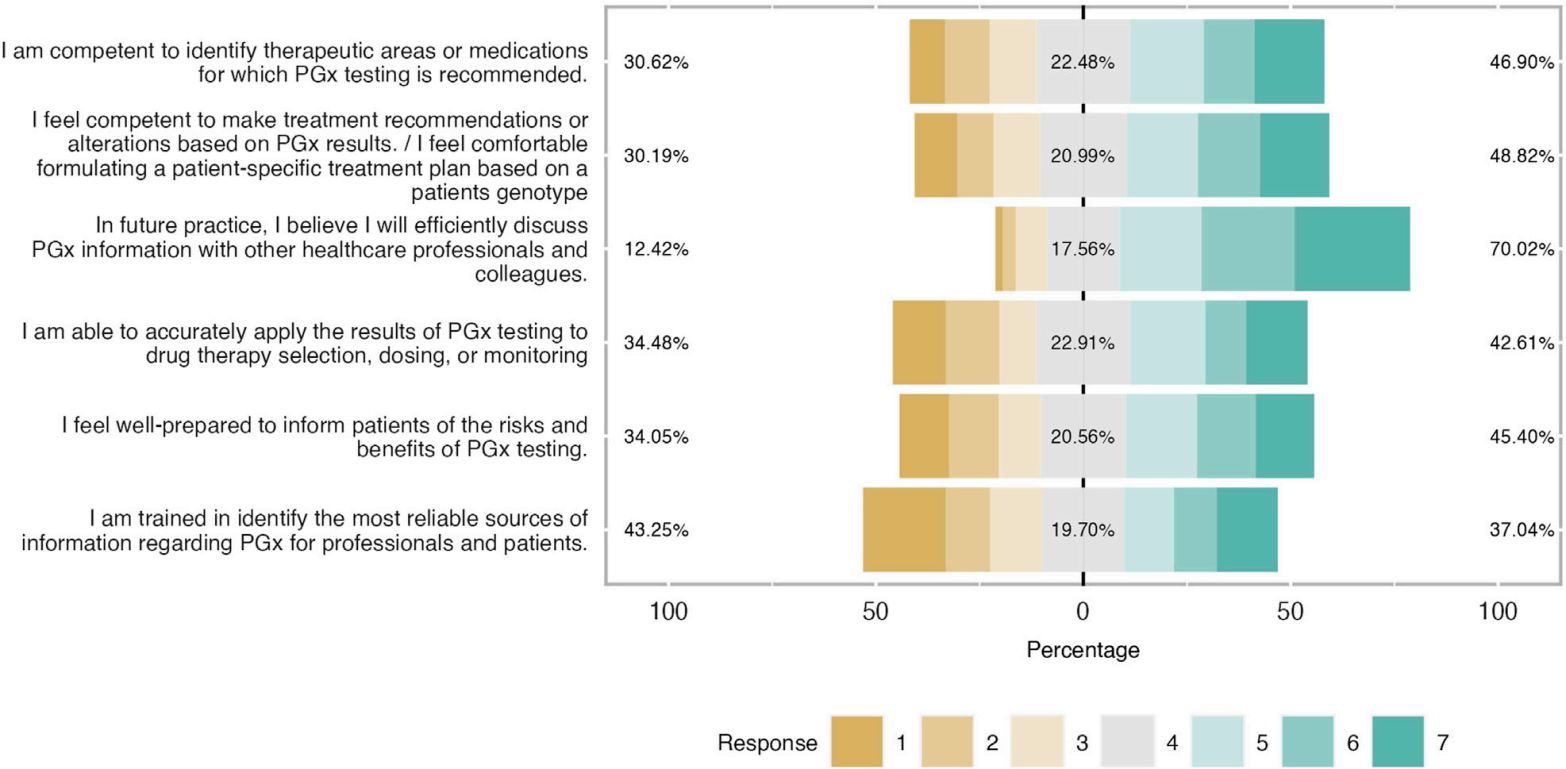
Students’ self-confidence towards PGx clinical application expressed in percentages. Respondents showed a good self-confidence and were prepared to implement I in ther future career.

**FIGURE 7:**
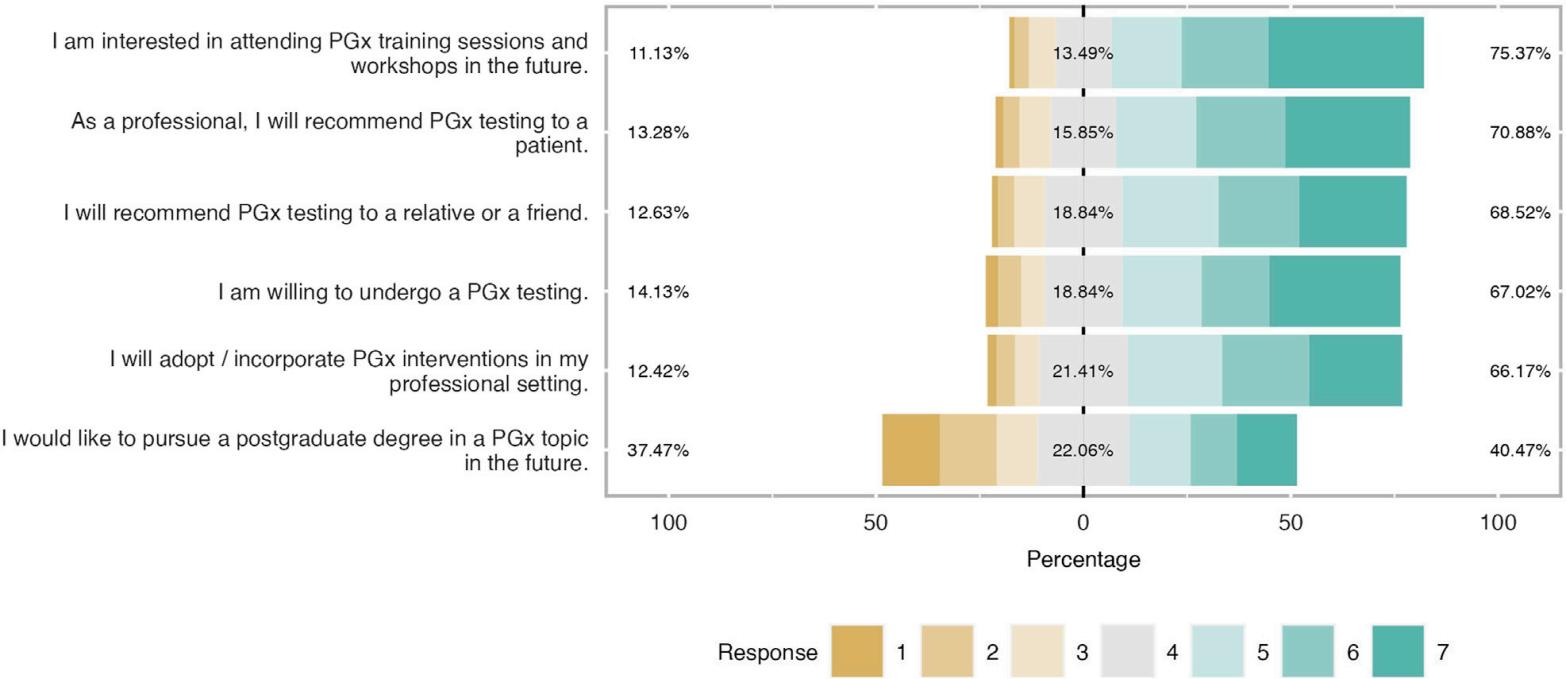
Students’ willingness to adopt PGx in the future. The majority of respondents were prone to recommend PGx testing in their family or patient while 67% will implement PGx testing in their future career. However, one-third of students were not interested in continue their studies in PGx.

This study’s results are shown in Tables 2; Figures 1–7. Cronbach analysis was performed. As demonstrated in Table 2 four out of the five factors of this study’s instrument had a Cronbach’s alpha value above 0.8. Only level of knowledge had a Cronbach’s alpha value of 0.509. Given the fact that Cronbach’s alpha coefficient measures the internal consistency of a scale and a coefficient of 0.7 or higher is generally considered acceptable, it is indicated that the overall internal consistency of the questionnaire scale is acceptable. The level of knowledge as a factor is less consistent probably due to the different measuring scales used.

**TABLE 2:** Cronbach’s alpha values for each section.

Sections	Cronbach’s Alpha
Section B= Knowledge	.509
Section C=Attitudes	.851
Section D = Self-confidence	.875
Section E = Intention to Adopt	.858
Section F=Barriers	.812

Upon confirming instrument validity, a confirmatory factor analysis was performed, as shown in Tables 3, 4, 5, close to 0.5 indicating that the proposed model has an acceptable fit. However, GFI, NFI, and RFI indexes were close to 0.8 but not close to 0.9, a fact that highlights that the model proposed is not the perfect fit. The model is applied in a highly diverse cohort in terms of nationalities, scientific backgrounds, and years of studies, and thus, having at least a few parameters within acceptable ranges, it can be concluded that the model is of good fit (Doll et al., 1994; Baumgartner and Homburg, 1996).

**TABLE 3:** Results of factor analysis.

Model	NPAR	CMIN	DF	*p*-value	CMIN/DF	GFI	AGFI	PGFI	NFI Delta 1	RFI rho1	TLI rho2	CFI
Default Model	82	1726.11	584	.000	2.956	.793	.764	.696	.762	.743	.814	.827
Saturated model	666	.000	0			1.000			1.000			1.000
Independence model	36	7246.36	630	.000	11.502	.282	.241	.267	.000	.000	.000	.000

AGFI: adjusted goodness of fit index, CFI: comparative fit index, CMIN: Chi-square statistics, DF:/degree of freedom, GFI: goodness of fit index, NFI: normed fit index, PGFI: parsimony goodness of fit index, RFI: relative fit index, TLI: Tucker–Lewis index.

**TABLE 4:** Results of factor analysis (RMSEA).

Model	RMSEA	LO 90	HI 90	PCLOSE
Default model	.065	.061	.068	.000
Independence model	.150	.147	.153	.000

RMSEA: root mean square error of approximation, LO, 90 = Lower than 90, HI, 90 = higher than 90.

**TABLE 5:** Model’s fitness check.

Model Summary				
Model	R	R Square	Adjusted R Square	Std. Error of the Estimate
1	,769^a^	,591	,587	,80,035

### Regression analysis

Moreover, to investigate the interaction between items and factors, a multiple regression analysis was conducted. Most of the item estimates (items related to demographics were not included in the analysis) were found to be close to 0.7. The coefficient of determination (R^2^)

was 0.769 and R^2^ was close to 0.6, signifying that the model was a good fit. Items about the level of knowledge were less than 0.6. A positive R-value indicates that a linear relationship exists between the dependent variable and the independent variables, while a negative R-value pinpointed an inverse relationship between them. The absolute value of R indicates the strength of the relationship. R^2^ is an indicator of the proportion of the variability of the dependent variable that is inevitably explained by the independent variables in multiple regression analysis.

In the current regression analysis, the standardized beta coefficients for factors level of knowledge, attitudes, self-confidence, barriers, and concerns were found to be 0.050, 0.514, 0.243, and 0.147, respectively. A positive beta coefficient indicates that the dependent variable will increase as the independent variable increases. Therefore, it was found that attitudes exert the greatest effect on students’ intentions to adopt PGx, followed by barriers and concerns in PGx adoption and their level of self-confidence. The level of knowledge appeared not to exert a significant impact. It is not clear whether this observation is confirmed since this factor was measured on a different scale. Furthermore, the effect of attitude is the most per the standardized regression weight suggesting that there is a strong positive impact of the factor on students’ intentions. Barriers and self-confidence shared a similar effect and less than that of attitudes, while the level of knowledge had a very low effect. Correlation between items and factors was very high showing a great fit (see Supplementary Tables S1–3). The level of knowledge is positively correlated with attitudes with an estimate of 0.541 and barriers are positively correlated with attitudes as a factor with an estimate of 0.679.

### Attitudes

Students shared an overall positive attitude about PGx testing in the clinical practice and it was demonstrated that they were aware of its clinical benefits. More precisely, 82% of students agreed that PGx testing will improve drug efficacy and optimize drug dosage, while the majority of them (around 70%) agreed that will lead to a significant decrease in the incidence rate of ADRs and improve patients’ quality of life during a drug therapy. The same trend was observed when respondents were asked about PGx’s role in medication expenditures since almost 80% pointed out that agreed with this item and 17% had a neutral opinion. Finally, two-thirds of respondents considered PGx relevant to their professional setting and the same proportion believed that part of their professional role is to counsel patients regarding PGx information (Figure 2). When respondents’ answers were analyzed based on their gender, it was demonstrated that both male and female students demonstrated a similar positive attitude and there were only slight differences in the two items. Indeed, female students presented a more positive attitude by 12% compared to their male counterparts when they were asked about the relevance of PGx in their profession and they were more convinced that more patients will undergo PGx testing in the future (Figure 3).

### Level of knowledge

Furthermore, the level of knowledge was also investigated. Students were found to have a moderate to good level of knowledge. Almost 60% of the respondents answered correctly the relevant questions especially those related to general theoretical knowledge. This trend is not followed when the question is more specific and lab-based (Figure 4). Two-thirds of participants gave the wrong answer when they were asked if genetic determinants of drug response change over a person’s lifetime. Almost half of the participants were not sure if PGx testing is available for all medications and that the gene is involved in warfarin metabolization. More precisely, 23% were neutral and only 9% found the correct answer.

### Barriers and concerns

Participants were aware of the most cited barriers and concerns related to PGx testing implementation as shown in Figure 5; Table 6. Indeed, the most important barriers based on the respondents’ feedback were the lack of trained personnel (79%), followed by the cost of PGx testing (73%), data privacy concerns (64%), and the lack of reimbursement. A great percentage of participants (54%) believed that PGx can cause psychological distress to patients (mean = 4.63 and SD = 1.63). Finally, students did not consider the existence of moral and religious concerns as a very significant barrier (mean 4.38 and SD = 1.77). Only 44% agreed on that while 29% were neutral and 27% were negative.

**TABLE 6:** Mean and Standard Deviation of Items used in the study’s questionnaire.

	Mean	SD^a^
Attitudes 1	5.25	1.48
Attitudes 2	5.86	1.29
Attitudes 3	5.22	1.44
Attitudes 4	5.3	1.55
Attitudes 5	5.71	1.27
Attitudes 6	5.62	1.36
Self-Confidence 1	4.34	1.84
Self-Confidence 2	4.38	1.88
Self-Confidence 3	5.29	1.52
Self-Confidence 4	4.09	1.91
Self-Confidence 5	4.18	1.91
Self-Confidence 6	3.83	2.05
Willngness to adopt 1	5.54	1.53
Willngness to adopt 2	4.02	1.95
Willngness to adopt 3	5.21	1.67
Willngness to adopt 4	5.11	1.51
Willngness to adopt 5	5.2	1.5
Willngness to adopt 6	5.32	1.56
Barriers 1	5.53	1.42
Barriers 2	5.6	1.35
Barriers 3	4.38	1.77
Barriers 4	5.1	1.59
Barriers 5	5.11	1.5
Barriers 6	5.09	1.62
Barriers 7	4.63	1.63

^a^ Standard Deviation.

### Self-confidence

When students were asked to characterize their self-confidence in other words, to describe their readiness and capability in practicing PGx in their future clinical practice, it was stated as moderate as it is illustrated in Figure 6. Indeed, it was found that around 50% of participants were competent to identify therapeutic areas or medications with PGx recommendations and almost half of the respondents stated to be comfortable to formulate a patient’s treatment scheme based on PGx results (mean = 4.34 and SD = 1.84). The vast majority believed that they would efficiently discuss PGx testing information with their healthcare colleagues. Their level of readiness dropped when it came to implementing PGx results in drug therapy selection, dosing, and monitoring whereas a third of students did not feel well-prepared to inform patients about the benefits and risks of PGx testing (mean = 4.09 and SD = 1.91). Finally, students were not shown to be confident about their educational training to identify the proper source of clinical information about this topic. Indeed 43% claimed not to be well trained, 20% had a neutral position and only 37% were positive (mean = 3.83 and SD = 2.05).

### Intention to adopt PGx in the clinical practice

As shown in Figure 7, participants were willing to incorporate PGx testing in their clinical practice in the future. There was an interest in expanding their knowledge and expertise in the topic either by pursuing a postgraduate program or attending a seminar. Admittedly, almost 60% of the students (mean = 5.54 and SD = 1.53) would like to attend a workshop or a PGx training in the future while 41% were prone to pursue a PGx-related postgraduate program (mean = 4.02 and SD = 1.95). The majority of students (almost 70%) were willing to conduct a PGx test for themselves (mean 5.21 and SD = 1.67), and 67% were positive in recommending it to a relative or a friend. (mean = 5.20 and SD = 1.50). It was also noticed that respondents had a positive tendency to apply PGx testing in their clinical routine because more than half of them (67% and 70% respectively) answered that they would implement it in their professional setting in the future and they would recommend it to a patient (mean = 5.11 and SD = 1.51 and mean = 5.32 and SD = 1.56).

## Discussion

PGx is an emerging field of personalized medicine that can offer a series of advantages in drug management. Besides the plenty and proven clinical benefits, the adoption rate of PGx applications in clinical practice remains low across the globe. The main way to boost PGx implementation is by investing in having adequately trained future generations of healthcare professionals. For this reason, we aimed to investigate the attitudes, beliefs, and level of knowledge of health science undergraduate students of UoS.

According to our findings, the proposed model had a good internal consistency since four out of five independent factors had a Cronbach α that was over 0.8. The model had also a good fit (CMIN/DF was almost three while RMSEA was found at 0.65), a fact that is highly important since in social studies it is not common to find high consistency scores. In addition, the proposed model managed to fit the multinational and heterogeneous character of the sample, a fact that is also significant. Based on the regression analysis, attitude was the factor with the greatest impact on students’ intentions to adopt PGx which also correlated with barriers and level of knowledge. The level of knowledge did not fit well in the model and had a meaningless impact on students’ intentions. Self-confidence and barriers were shown to contribute to students’ willingness to adopt, while barriers were positively correlated with attitudes as well.

Moreover, UoS students had a positive attitude, a moderate level of knowledge along with good level of self-confidence. Most of the respondents confirmed that PGx is relevant to their profession whereas, as it was indicated, a great percentage of students were aware of PGx clinical applications and its benefits. There is a slight difference between students’ attitudes based on their gender, while, they considered that lack of reimbursement, high PGx testing cost, shortage of trained personnel, and data privacy concerns were the main obstacles to slow PGx adoption. The majority of students were willing to broaden their knowledge in the field via a postgraduate course or a seminar. In addition, they intend to apply PGx testing in their clinical routine in the future and most of them would recommend a relevant test to a patient.

Based on the literature, undergraduate students who attend health-related courses in medicine, pharmacy, or nursing share a positive attitude toward PGx applications. In Wen and coworkers, 2022 study, it was shown that the vast majority of first-year pharmacy students in the United States considered PGx as a useful tool and 57% agreed that it is relevant to their profession while 22% totally agreed that PGx will be relevant to their clinical practice (Wen et al., 2022). Siamoglou and coworkers, 2021 also concluded with similar results. Students from Malaysia and Greece shared a positive attitude towards genetic testing and were aware of the benefits and relative advantages of preemptive testing (Siamoglou et al., 2021a). Finally, according to Shah and coworkers, 2022, female pharmacy students in Pakistan demonstrated a better attitude towards PGx testing compared to their male counterparts, an observation that comes in agreement with our results (Shah et al., 2022).

Most of the available publications indicated that undergraduate students showed a weak or moderate level of knowledge (Siamoglou et al., 2021b; Koufaki et al., 2022; Makrygianni et al., 2023). According to Makrygianni and coworkers, 2023, this factor did not exert any impact on students’ intentions (Makrygianni et al., 2023). Koufaki and coworkers, 2022 concluded that Malaysian and Greek pharmacy students had a rather low level of knowledge (Koufaki et al., 2022). Furthermore, Arafah and coworkers, 2022 mentioned that the overall level of knowledge of Saudi Arabian pharmacy students was low and that students lacked practical skills, an observation that was also made by Makrygianni and coworkers, 2023 (Arafah et al., 2022; Makrygianni et al., 2023). Makrygianni and coworkers, 2023, also presented that students were reluctant to answer advanced technical questions, and that had an impact on their self-confidence (Makrygianni et al., 2023). Finally, graduate pharmacy students expressed that gaining in-depth knowledge was a key to their future career advancement based on Koufaki and coworkers, 2023 (Koufaki et al., 2023).

Moreover, the level of knowledge was positively correlated with attitudes, an observation that agrees with the existing literature (Makrygianni et al., 2023). However, it was demonstrated that the moderate level of knowledge has not negatively affected students’ attitudes or self-confidence. Students have a high level of self-confidence about implementing PGx in the future and only items related to training readiness received less positive feedback, an observation that might be due to the level of knowledge. This is congruent with other studies (Mehtar et al., 2022 and Domnic et al., 2022). For instance, based on Mehtar and coworkers 2022, in Lebanon, approximately, 73% of all pharmacy students stated that they should be able to identify patients that might benefit from any type of genetic testing and they could use PGx, in their future practice (Mehtar et al., 2022). Regarding students’ reluctance in being able to adjust or alter a patient’s treatment following PGx testing, Domnic, and coworkers, 2022 study, pointed out that 36% of medical students agreed that they were confident to use the PGx results to stratify a patient’s treatment, whereas 40% agreed that they needed better knowledge (Domnic et al., 2022). This result comes per our findings.

Furthermore, barriers and concerns were indicated to be an influential factor and may determine students’ intentions to adopt PGx in their future clinical profession, especially those related to PGx testing cost, lack of reimbursement, and data privacy issues. Based on the literature, data privacy and results’ confidentiality are the most cited issues. Indeed, some studies pinpointed that the main concerns refer to data privacy and results’ confidentiality and others focused more on PGx logistics including costs, lack of trained personnel, and lack of complete clinical guidelines (Bank et al., 2018; Cheung et al., 2021). In the Netherlands, Bank and coworkers, 2018 showed that 72% of the participants expressed their concern about data use and the chance of being provided to unauthorized individuals while 88% believed that PGx testing results could provoke psychological distress to patients (Bank et al., 2018).

A study conducted by Cheung and coworkers, in 2021 had also come up with relevant results in Hong Kong (Cheung et al., 2021). Nonetheless, Koufaki and coworkers, 2022 stated that Greek students worried more about PGx cost and the lack of complete clinical guidelines while their Malaysian counterparts were concerned about data privacy (Koufaki et al., 2022). In the aforementioned study, it was implied that the difference between the two students’ cohorts was the cultural context because the local legislation and directives had affected students’ perceptions. In the present analysis, though, we did not notice extreme differences even if we investigated a highly diverse and multinational environment. The UAE is a cosmopolitan with diverse ethnicities from almost all over the world, working and studying together in an inclusive environment and respecting high standards of understanding and tolerance.

Finally, as far as respondents’ willingness to implement PGx in their professional lives, our findings come following the literature. Based on Arafah and coworkers, 2022 study, 61.2% of pharmacy students were interested in a PGx-related course or seminar (Arafah et al., 2022). The vast majority expressed an interest in participating in genetic research, and they were willing to undergo PGx testing, too (Arafah et al., 2022). Jarrar and coworkers, 2019, concluded with similar results; around 93% of pharmacy students were willing to learn more about PGx testing, whereas 31% opted to pursue a postgraduate program in the field (Jarrar et al., 2019). Finally, in a study that was conducted among professional pharmacists and pharmacy students in Lebanon, 62% of participants were interested in learning more about PGx while in Croatia, the majority of students (dental, medicine, pharmacy) were willing to undergo a PGx test, a finding that is close to our results (Bukic et al., 2022; Mehtar et al., 2022).

## Limitations

This study has a few limitations. The survey was conducted among undergraduate students of the University of Sharjah and it did not include any other UAE university. To overcome this limitation, surveys were distributed to four different colleges to broaden our research sample. Questionnaires were distributed online and not via direct contact. This fact might lead to response bias but it is not shown to have such case in our analysis. Furthermore, the response rate was estimated at 30%. This rate was low but the total sample is sufficient to get results with significant statistical power. Finally, students came from different academic background and this was not taken into consideration in the scope of this analysis to identify any differences.

## Conclusion

PGx is a hot topic of personalized medicine with great clinical applications. Implementation of PGx in healthcare systems remains a major challenge. The adoption rate of PGx is quite low worldwide and also in the UAE. A key factor for expanding PGx application in the clinical practice is based on the involvement of healthcare professionals. The future generations of UAE healthcare professionals in this study were shown to be aware of PGx and had a good level of knowledge. Their attitude towards PGx was positive and a great percentage of them planned to incorporate PGx testing in the clinical routine, while they were more than willing to undergo a relevant test for themselves. Moreover, the respondents expressed their opinions and concerns about the most commonly shared barriers and challenges related to PGx testing. The cost of PGx testing, lack of specialized personnel, and data confidentiality were found to be the most important challenges for PGx clinical implementation. In future research, the impact of demographics including gender, academic background, and year of study on students’ intentions to adopt PGx in clinical practice will be investigated further.

## Data Availability

The original contributions presented in the study are included in the article/Supplementary Material, further inquiries can be directed to the corresponding author.

## Abbreviations

ADR: Adverse Drug Reaction; AGFI: Adjusted Goodness of Fit Index; CFA: Comparative Factor Analysis; CFI: Comparative Fit Index; CMIN/DF: Chi-square statistics/degree of freedom; GFI: Goodness of Fit Index; NFI: Normed Fit Index; PGx: Pharmacogenomics; PGFI: Parsimony Goodness of Fit Index; RFI: Relative Fit Index; RMSEA: Root Mean Square Error of Approximation; SD: Standard Deviation; TLI: Tucker–Lewis index; TPB: Theory of Planned Behavior; UAE = United Arab Emirates; UoS = University of Sharjah.
