# Supplementary material for: Do future healthcare professionals advocate for pharmacogenomics? A study on medical and health sciences undergraduate students
Source: Front Pharmacol. 2024 Apr 11;15:1377420. doi: 10.3389/fphar.2024.1377420 (PMC11043592; doi:10.3389/fphar.2024.1377420)
Supplement: Supplementary file 1 [file DataSheet1.PDF]

Do Future Healthcare Professionals advocate for Pharmacogenomics to achieve better healthcare outcomes? Awareness, Attitudes, and Intentions of Heath Sciences Undergraduate Students at University of Sharjah.

Supplementary Material

Sup Table 1: Standardized Regression Weights.

			Estimate	S.E.	C.R.	P
Intentions	<---	Knowledge	.223	.234	.950	.342
Intentions	<---	Barriers	.161	.072	2.237	.025
Intentions	<---	Attitudes	**.611**	.072	8.502	***
Intentions	<---	Self-confidence	.145	.030	4.846	***
Knowledge 11	<---	Knowledge	1.000			
Knowledge 10	<---	Knowledge	-.427	.193	-2.218	.027
Knowledge 9	<---	Knowledge	1.085	.175	6.206	***
Knowledge 8	<---	Knowledge	.576	.144	4.000	***
Knowledge 7	<---	Knowledge	1.322	.211	6.273	***
Knowledge 6	<---	Knowledge	1.023	.173	5.922	***
Knowledge 5	<---	Knowledge	1.146	.194	5.909	***
Knowledge 4	<---	Knowledge	1.089	.172	6.334	***
Knowledge 3	<---	Knowledge	.291	.169	1.722	.085
Knowledge 2	<---	Knowledge	-.311	.172	-1.809	.070
Knowledge 1	<---	Knowledge	.399	.099	4.032	***
Barriers 1	<---	Barriers	1.000			
Barriers 2	<---	Barriers	1.015	.096	10.589	***
Barriers 3	<---	Barriers	1.070	.119	9.001	***
Barriers 4	<---	Barriers	1.335	.117	11.417	***
Barriers 5	<---	Barriers	1.248	.110	11.336	***
Barriers 6	<---	Barriers	1.244	.116	10.757	***
Barriers 7	<---	Barriers	1.048	.111	9.405	***
Attitude 6	<---	Attitude	1.000			
Attitude 5	<---	Attitude	.907	.050	18.059	***
Attitude 4	<---	Attitude	.953	.064	14.989	***
Attitude 3	<---	Attitude	.889	.059	15.071	***
Attitude 2	<---	Attitude	.873	.052	16.752	***
Attitude 1	<---	Attitude	.794	.062	12.785	***
Self-confidence 6	<---	Self-confidence	1.000			
Self-confidence 5	<---	Self-confidence	.973	.058	16.719	***
Self-confidence 4	<---	Self-confidence	1.038	.058	17.845	***
Self-confidence 3	<---	Self-confidence	.501	.047	10.570	***
Self-confidence 2	<---	Self-confidence	.943	.057	16.466	***
Self-confidence 1	<---	Self-confidence	.927	.056	16.511	***
Intentions 1	<---	Intentions	1.000			
Intentions 2	<---	Intentions	.920	.088	10.399	***

			Estimate	S.E.	C.R.	P
Intentions 3	<---	Intentions	1.101	.076	14.479	***
Intentions 4	<---	Intentions	1.153	.069	16.594	***
Intentions 5	<---	Intentions	1.106	.069	16.065	***
Intentions 6	<---	Intentions	1.165	.072	16.290	***
***p-value<0.05						

Sup. Table 2: Correlations between independent factors.

			Estimate
Knowledge	<-->	Barriers	.426
Knowledge	<-->	Attitudes	.541
Knowledge	<-->	Self-confidence	.081
Barriers	<-->	Attitudes	.679
Barriers	<-->	Self-confidence	.376
Attitudes	<-->	Self-confidence	.418

Sup. Table 3: Coefficients analysis.

					95.0% Confidence Interval for B						Collinearity Statistics	
	Unstandardized B	Coefficients St	Standardized Coefficients Beta	t	Sig.	Lower Bound	Upper Bound	Zero-order	Partial	Part	Tolerance	VIF
Constant	-.631	.378		-1.668	0.96	-1.375	.112					
Level of knowledge	.243	.157	0.5	1.549	.122	-0.65	.552	.320	0.72	0.46	.858	1.166
Attitudes	.603	0.44	.514	13.693	<0.001	.517	.690	.715	.537	.408	.629	1.591
Self-Confidence	.208	0.29	.243	7.205	<0.001	.151	.264	.533	.318	.214	.778	1.285
Barriers & Concerns	.172	0.43	.147	4.035	<0.001	0.88	.255	.529	.185	.120	.666	1.501

Do Future Healthcare Professionals advocate for Pharmacogenomics to achieve better healthcare outcomes? Awareness, Attitudes, and Intentions of Heath Sciences Undergraduate Students at University of Sharjah.

Supplementary Material 2

Section A
Demographic data

A1. Gender:
Male ☐ Female ☐

A2. Program

☐

A3. Degree

☐

A4. Year of studies:

1st	2nd	3rd	4th	5th	6th

A5. Have you attended any PGx-related lectures ?
Yes ☐ No ☐

Section B
Assessment of general knowledge in PGx interventions.
Answer the following questions based on your understanding by choosing agree, disagree or not sure.

	Agree	Disagree	Not Sure
Slight differences in a person's genome can have a major impact on how the person responds to a medication.			
PGx diagnostic testing is currently available for most medications.			
Genetic determinants of drug response change over a person's lifetime.			
PGx interventions can optimize drug dosing and increases drug efficacy.			
People's differences in terms of pharmacodynamics and the interaction between medication and molecular targets exists due to genetic variance.			
Drug effects can be altered by genetic variability in patients.			
The action of pharmacokinetic parameters like absorption, distribution, etc can be influenced by person's genetic makeup.			

A person's response to warfarin is affected by genetic variance in ***CYP2C9*** and ***VKORC I*** gene.			
PGx will improve health care due to the optimization of medication prescription, early disease prognosis, reduction of healthcare expenditures.			
PGx doesn't play an important role in identifying drug-drug interactions.			
PGx reduces the occurrence and severity of adverse event and rationalized medication prescription.			

Section C

Attitudes and Perceptions towards PGx and its implications

What are your main thoughts about PGx matters? Please fill out each option: 1= totally disagree to 7=totally agree

	**Totally Disagree**			**Neutral**			**Totally Agree**
PGx is relevant to my professional setting.	**1**	**2**	**3**	**4**	**5**	**6**	**7**
PGx testing will improve drug efficacy and optimize drug dosing.	**1**	**2**	**3**	**4**	**5**	**6**	**7**
Patients will be urged to undergo a PGx testing in the future.	**1**	**2**	**3**	**4**	**5**	**6**	**7**
Part of my professional role should include counseling patients regarding PGx information.	**1**	**2**	**3**	**4**	**5**	**6**	**7**
PGx testing will lead to a significant decrease to the incidence rate of adverse drug reactions and improve patient's quality of life during a drug therapy.	**1**	**2**	**3**	**4**	**5**	**6**	**7**
PGx testing can improve the control of drug therapy expenditures.	**1**	**2**	**3**	**4**	**5**	**6**	**7**

Section D

Confidence/ self -efficacy in applying PGx in a professional setting

Rate your self-efficacy in PGx topics (1= totally disagree - 7=totally agree).

	**Totally Disagree**			**Neutral**			**Totally Agree**
I am competent to identify therapeutic areas or medications for which PGx testing is recommended.	**1**	**2**	**3**	**4**	**5**	**6**	**7**
I feel competent to make treatment recommendations or alterations based on PGx results./ I feel comfortable formulating a patient-specific treatment plan based on a patient's genotype.	**1**	**2**	**3**	**4**	**5**	**6**	**7**
In future practice, I believe I will efficiently discuss PGx information with other healthcare professionals and colleagues.	**1**	**2**	**3**	**4**	**5**	**6**	**7**
I am able to accurately apply the results of PGx testing to drug therapy selection, dosing, or monitoring.	**1**	**2**	**3**	**4**	**5**	**6**	**7**
I feel well-prepared to inform patients of the risks and benefits of PGx testing.	**1**	**2**	**3**	**4**	**5**	**6**	**7**

I am trained in identify the most reliable sources of information regarding PGx for professionals and patients.	**1**	**2**	**3**	**4**	**5**	**6**	**7**

Section E

Willingness to adopt PGx in clinical practice and future perspectives

What are your plan for the future about PGx m? Please fill out each option: 1= totally disagree to 7=totally agree.

	**Totally Disagree**			**Neutral**			**Totally Agree**
I am interested in attending PGx training sessions and workshops in the future.	**1**	**2**	**3**	**4**	**5**	**6**	**7**
I would like to pursue a postgraduate degree in a PGx topic in the future.	**1**	**2**	**3**	**4**	**5**	**6**	**7**
I am willing to undergo a PGx testing.	**1**	**2**	**3**	**4**	**5**	**6**	**7**
I will adopt/ incorporate PGx interventions in my professional setting.	**1**	**2**	**3**	**4**	**5**	**6**	**7**
I will recommend PGx testing to a relative or a friend.	**1**	**2**	**3**	**4**	**5**	**6**	**7**
As a professional, I will recommend PGx testing to a patient.	**1**	**2**	**3**	**4**	**5**	**6**	**7**

Section F

Barriers and Concerns in PGx implementation in clinical practice

What are the potential barriers in PGx implementation in clinical practice? Give your feedback by rating the below options from 1=totally disagree to 7=totally agree.

	Totally disagree			Neutral			Totally Agree
Do you think that PGx testing cost is a significant issue?	**1**	**2**	**3**	**4**	**5**	**6**	**7**
Do you believe that the lack of specialized personnel can cause delays in PGx implementation in clinical practice?	**1**	**2**	**3**	**4**	**5**	**6**	**7**
Do you think that there are moral and religious issues which impede PGx adoption in clinical practice?	**1**	**2**	**3**	**4**	**5**	**6**	**7**
Is data safety and privacy an important barrier?	**1**	**2**	**3**	**4**	**5**	**6**	**7**
The fact that PGx testing isn't reimbursed by most healthcare systems causes a problem.	**1**	**2**	**3**	**4**	**5**	**6**	**7**
Are you worried that PGx test's results could be passed to an unauthorized person?	**1**	**2**	**3**	**4**	**5**	**6**	**7**
Do you think that PGx testing could cause a patient psychological distress?	**1**	**2**	**3**	**4**	**5**	**6**	**7**
